# Comparison of mini-open, anteroinferior psoas approach and mini-open, direct lateral transpsoas approach for lumbar burst fractures: A retrospective cohort study

**DOI:** 10.3389/fsurg.2022.995410

**Published:** 2022-10-14

**Authors:** Bin Pan, Weiyang Yu, Chao Lou, Jiawei Gao, Wenjun Huang, Dengwei He

**Affiliations:** Department of Orthopedics, Lishui Hospital, Zhejiang University School of Medicine, Lishui, China

**Keywords:** anteroinferior psoas, lateral transpsoas approach, lumbar burst fractures, mini-open, posterior/anterior combined surgery

## Abstract

**Objective:**

We evaluated the effect of a novel modified OLIF technique (anteroinferior psoas approach, AIPA) for anterior decompression reconstruction in lumbar burst fractures, and compared the clinical, radiological outcomes and approach-related complications with the mini-open, lateral transpsoas approach (LTPA).

**Methods:**

From March 2016 to November 2019, 68 patients with lumbar burst fractures underwent one-stage monosegmental posterior/anterior surgery from L1–L4 segments. 35 patients included in AIPA and 33 patients in LTPA group underwent anterior decompression reconstruction. The clinical, radiological and functional evaluation outcomes were recorded during the 16–60 months follow-up period.

**Results:**

At the latest follow up, neurological state of one or more ASIA grades were achieved in AIPA (90.9%) and LTPA group (94.9%). No significant differences were noted between the two groups regarding preoperative and postoperative Cobbs angle. The surgery time (192.29 vs. 230.47 min, *P *= 0.02) in AIPA group was better compared with LTPA. The AIPA showed better improvement on Oswestry Disability Index (43.4% vs. 60.8%, *P *< 0.05) and Mental Component Score (49.0% vs. 43.7%, *P *< 0.05) one month after surgery, but no difference at the latest follow-up. 10 patients (9 in LTPA and 1 in AIPA) experienced temporary motor deficits in hip flexor and groin or thigh numbness, which disappeared six months after surgery.

**Conclusions:**

Compared with lateral transpsoas approach, anterior decompression reconstruction *via* mini-open, anteroinferior psoas approach was a safe and less invasive approach, with fewer approach-related complications in the treatment for unstable lumbar burst fractures

## Introduction

Burst fractures are typically caused by motor vehicle accidents or falls from heights, accounting for 21%–58% of all types of thoracolumbar fractures (1). Anatomically, burst fractures are characterized by the fracture of the anterior and middle columns with or without the posterior column of the spine. The surgical approaches for lumbar burst fractures include posterior, anterior, lateral, or combined approaches (2–5). Anterior approaches have been reported in previous studies as an effective strategy for unstable thoracolumbar burst fractures (6, 7); Anterior approaches for corpectomy and titanium cage reconstruction were performed to directly decompress the neural elements and restore biomechanical support (5).

The complications of the traditional anterior open transperitoneal approach, including vascular injury, postoperative bowel obstruction, retrograde ejaculation, and incisional hernia, have been widely reported (8). Mini-open extreme lateral transpsoas approach (LLIF/LTPA), which was devised for lumbar degenerative diseases (9), has been preliminarily applied for corpectomy and anterior reconstruction in thoracolumbar burst fractures (10). Gurpreet and Eck et al. reported that adequate visualization of the spinal canal and decompression was achieved without massive sequelae to vascular or neural tissue (11). However, the risk of the psoas and lumbar plexus injury could not be ignored. With a continuous increase in the use of the LTPA technique, the approach-related complications increased, which usually were caused by prolonged surgery time, increasing retractor utilization, and direct mechanical injury (10, 12, 13). Although sensory and motor-related complications of LTPA for corpectomy have been reported, further systematic studies are still lacking.

Oblique lateral interbody fusion (OLIF), which was designed to access the disc space *via* entering the anatomical space between the psoas and the aorta, has been the proposed a solution to the approach-related disadvantages of ALIF and LLIF (14). Compared with LLIF, OLIF reduces the risk of lumbar plexus injury (15). However, it is still not safe to settle the surgical tube system using just the physiological gap between major vessels and the psoas (16). The diameter of the OLIF dilation tube or PEEK cage was a little bigger than the anatomical access corridor in some cases (17).

The anteroinferior psoas (AIP) approach, which is a modified direct visualization lateral approach of OLIF (Oblique Lumbar Interbody Fusion), aims to decrease psoas and lumbar plexus injury and especially operate under direct visualization compared with the OLIF approach. Briefly, the psoas fascia was dissected from the surface of the lumbar disc using a Cobb dissector under direct visualization. Subsequently, the psoas was retracted posteriorly with retractor to obtain an adequate view. Fan and Hu et al. first reported the use of this technique for treating lumbar degenerative diseases and proved its safety and efficacy (16, 18). Since the mild retraction of the psoas muscle can make the corridor obviously enlarged (17), AIP seems to provide greater decompression channels compared with the conventional OLIF channel. We applied the AIP technique for anterior decompression and titanium cage placement in unstable lumbar burst fractures and achieved promising results. This study aimed to detail the AIPA technique in treating lumbar burst fractures and compared the surgical results, radiological parameters, and functional scores with conventional LTPA.

## Materials and methods

### Patients

**Figure 1:**
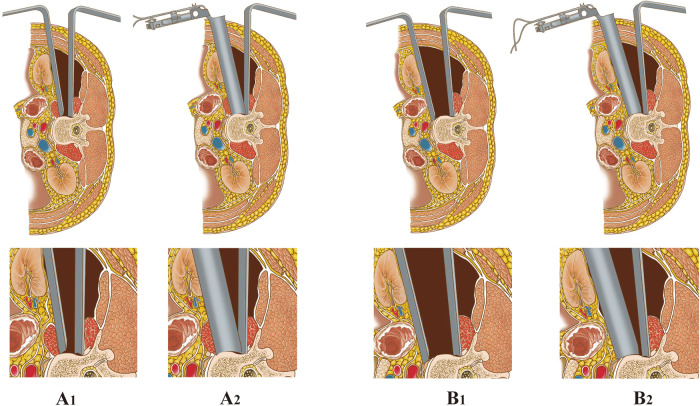
Two techniques for placement of anterior retractors *via* a mini-open incision. (**A_1−2_**) *LTPA*: The psoas was identified and bluntly split along the direction of the muscle fiber using a blunt-split device. Then, the retractor system was placed and fixed to the superior and inferior segments of the fractured vertebra. (**B_1−2_**) *AIPA*: Psoas muscle was retracted posteriorly from the border of the psoas; then, the retractor system was placed obliquely, then turned laterally and fixed to the superior and inferior segments of the fractured vertebra.

This study was reviewed and approved by the medical ethics committee of the author's hospital (Lishui Hospital Affiliated to Zhejiang University). From March 2016 to November 2019, 68 patients (48 male and 20 female) with lumbar burst fractures underwent one-stage posterior/anterior combined surgery. The stabilization of the vertebrae was supported by posterior percutaneous pedicle screw fixation first. Then, 33 patients (March 2016 to March 2018) were operated *via* a mini-open, extreme LTPA, and 35 patients (January 2018 to November 2019) were operated *via* AIPA for anterior decompression and titanium cage placement (Figure 1). The average time from injury to surgery was 3.1 days.

All patients were followed up until March 31, 2021. The shortest and longest follow-up periods were 16 months and 60 months, respectively. The inclusion criteria were as follows: age less than 65 years; single-level type-A3/A4 (posterior wall involvement) fractures classified by AO type with segmental instability or type-B/C (tension band injuries/displacement injuries) fractures (19); vertebral fracture levels selected from L1 to L4; and the load-sharing score >6. Several patients were excluded from the study due to osteoporotic (*T* value ≤ −2.5) or pathological fractures, severe multiorgan injuries requiring surgery, or previous history of spine surgery. The instability of the burst vertebra was described as sagittal vertebral body height loss >50%, local kyphosis deformity >20°, or comminution of the fractured vertebrae (20).

### Clinical data

The clinical data included surgery time, estimated blood loss, hospital stays, and postoperative neurological complications. Low back pain and physical function were assessed using the Oswestry Disability Index (ODI) score. Physical pain was also evaluated using the visual analogue scale (VAS) from 0 to 10 preoperatively and postoperatively. The neurological status was evaluated using the American Spinal Injury Association (ASIA) score. A 12-Item Short-Form Health Survey (SF-12) form (21) consisted of the physical component score (PCS) and mental component score (MCS), reflecting the information regarding the physical and mental statuses of a patient, respectively.

### Radiological data

The radiological data, including kyphotic angle, were measured between the superior endplate of the vertebra above the fractured vertebra and the inferior endplate of the vertebra below the fractured vertebra on the lateral radiograph (22). All measurements were performed by two experienced doctors not involved in this study. All radiographic evaluations were obtained three times by one of the two doctors, with the arithmetic mean recorded as the data of the present study.

### Statistical analysis

All statistical analyses were performed with the SPSS software 20.0 (IBM Statistics SPSS 20). The data were shown as mean ± standard deviation. The independent-two-sample *t* test or the Mann–Whitney *U* test was used to compare the difference between preoperative and postoperative results. The demographic data were compared using the chi-squared test and Fishers exact test. The significance was set at *P *< 0.05.

### Surgical technique

#### Posterior spinal surgery

After admission to the hospital, preoperative examinations including x-ray, Dual Energy x-ray Absorptiometry (DEXA), Computerized Tomography (CT) and Magnetic Resonance Imaging (MRI) were completed. The patient was placed in a prone position. Briefly, four small incisions, approximately 1.5 cm in length, were made in the skin projection of the pedicles. Two pedicle screws were inserted into each of the upper and lower vertebrae adjacent to the injured vertebra under C-arm x-ray monitoring. For type-C fracture, four pedicle screws were inserted into the cephalic and caudal vertebrae of the injured vertebra for additional stability. Next, two pre-bent longitudinal connecting rods were inserted into the U-shaped slots of the screws under the paraspinous muscle. The fractured vertebral body was repositioned along the longitudinal axis of the connecting rod using a distraction and compression tool (CanWell Inc. Zhejiang, China), which was matched with percutaneous transpedicular systems (22). Finally, the cephalic and caudal screws were tightened to stabilize the injured vertebra and avoid further kyphotic deformity aggravation.

### Minimally invasive corpectomy and titanium cage reconstruction *via* mini-open, LTPA

To date, previous studies (WD Smith, JS Uribe, Jason C. Eck, and Gurpreet) have described the minimally invasive lateral transpsoas approach to access the vertebral body in detail (3, 5, 11, 23). Briefly speaking, the patient was placed and fixed with a tape in the lateral decubitus position on the right side for anterior surgery. A pillow was placed over the iliac crest to further increase the space between the ribs and the iliac crest. A 5- to 6-cm transverse skin incision was made at the left midaxillary line under the monitoring of a C-arm x-ray. Abdominal muscles were bluntly split along the direction of their fibers, including the external oblique, the internal oblique, the transversalis, and the transverse abdominal fascia, reaching the retroperitoneal space. Sometimes extensive or partial rib resection was needed to reach the retroperitoneal space when the fractured vertebra was L2 or above. The retractors and periosteal detacher were used to retract the retroperitoneal contents anteriorly and bluntly split the psoas and lumbar plexus carefully until the fractured vertebra was exposed. Then, the dilating tubes were placed in sequence. The lumbar plexus tended to lie in the posterior one third of the psoas muscle. Electrophysiological monitoring was used in all cases to avoid injury to these motor nerves.

A tubular retractor system (CanWell Inc. Zhejiang, China) was placed over the final dilating tube, and then the tubes were removed. Finally, the retractor's blades were opened and secured to the lower and upper vertebrae under lateral and anterior/posterior fluoroscopy. The fractured vertebra or cartilaginous endplate was removed, and the spinal canal was decompressed with a drill, osteotome, or ultrasonic bone scalpel combined with rongeurs. If the lower endplate and the disc were intact, the lower endplate was preserved for maximum segmental motion. The fractured vertebra was replaced with a titanium cage and autologous bone. The titanium cage was knocked in until fluoroscopy was confirmed. The surgical incision was closed layer by layer, and a 5- to 6-cm incision was left. The surgery time and the bleeding volume were recorded.

### Minimally invasive corpectomy and titanium cage reconstruction *via* mini-open, AIPA

**Figure 2:**
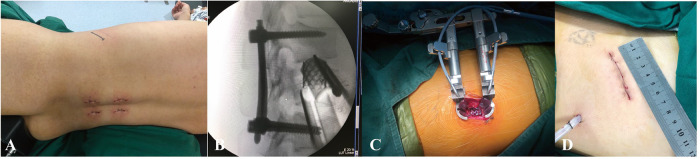
Operating room images demonstrating the AIPA surgical procedures. (**A**) Anterior incision of AIPA after posterior pedicle screw fixation. (**B**) Lateral radiograph demonstrating anterior titanium cage placement. (**C**) Position of the retractor system and the extent of exposure obtained. (**D**) Length of the anterior incision.

The surgeon evaluated the site of the abdominal aorta, vena cava, and sympathetic nerves in relation to the adjacency of the vertebra by the preoperative MRI first. After posterior percutaneous surgery, the anterior skin incision was made under fluoroscopy and marked to extend 2–3 cm along the margin of the indexed vertebra (16), which was slightly more anterior than the LTPA (Figures 1B, 2A). The surgical procedure to reach the retroperitoneal space using blunt separation instruments has been described above.

**Figure 3:**
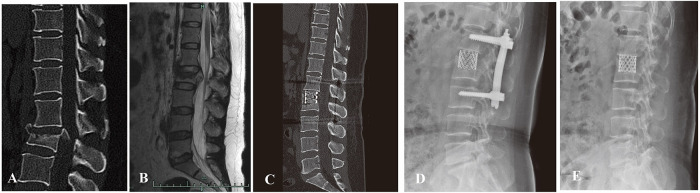
(**A,B**) preoperative sagittal CT scan and MRI images of a 42-year-old male patient with L2 burst fracture with nerve deficit. (**C**) Postoperative lateral CT image demonstrating good correction of kyphotic deformity *via* posterior instrumentation combined with AIPA anterior decompression and titanium cage placement. (**D**) An x-ray image was taken 3 months after surgery. (**E**) x-ray image demonstrating that the posterior screw–rod system had been removed.

Unlike the LTPA approach, AIPA does not require dissection of the psoas. The abdominal viscera, ureter, and vascular together with extraperitoneal fat were retracted anteriorly (ventral side) with a long retractor. The fascia of the anteroinferior border of the psoas was bluntly separated, and the psoas was retracted posteriorly (dorsal side) along the surface of the index vertebra and disc using Cobb dissector under direct visualization (Figure 1B). The probe, dilators and retractors were sequentially placed in an oblique direction and then the channel could turn laterally to fully expose the decompression area under direct visualization and facilitate the following surgical procedures. Finally, the retractor's blades were opened and secured to the lower and upper vertebrae under lateral and anterior/posterior fluoroscopy. The anterior decompression and titanium cage reconstruction were conducted as described above. The neuromonitoring equipment was not required during the procedure. The psoas and surrounding soft tissues were not or slightly damaged during the whole process (Figure 3).

## Results

A total of 68 patients, with 68 fractured segments, were included for treatment. The demographic data are listed in Table 1. No statistically significant differences in sex, age, mechanism of injury, TLIC score, and load-sharing score were found between the AIPA and LTPA groups. The modified AO spine classification (19) revealed the following: A3 (3), A4 (8), B1 (15), B2 (4), and C (3) in the LTPA group; A3 (4), A4 (6), B1 (18), B2 (5), and C (2) in the AIPA group.

**Table 1:** Demographic data of patients.

	AIPA	LTPA	*P* value*	*χ*2 value
Number of patients	35	33		
Age	48.9 ± 11.3	48.2 ± 9.6	0.85	
Sex ratio (M/F)	23/12	25/8	0.364	0.825
*Mechanism of injury*			0.724	0.647
Fall from height	18	20		
Motor accidents	11	9		
Other mechanism	6	4		
*Levels of fracture*			0.912	0.531
L1	8	6		
L2	14	12		
L3	8	9		
L4	5	6		
*AO Type*			0.917	0.954
A3	4	3		
A4	6	8		
B1	18	15		
B2	5	4		
C	2	3		
TLICs score	5.4 ± 1.5	5.6 ± 1.4	0.65	
Loading-Sharing score	8.1 ± 0.98	7.9 ± 1.2	0.55	

AIPA, anteroinferior psoas approach; LTPA, lateral trans-psoas approach; TLICS, Thoraco-Lumbar Injury Classification and Severity score.

* Significance was set at *P* < 0.05. The values are given as the mean ± the standard deviation.

### Clinical outcomes

The average surgery time in the LTPA group (230.47 ± 49 min) was significantly longer than that in the AIPA group (192.29 ± 34 min) (Mann–Whitney *U* test, *P* = 0.02). The mean blood loss during the surgery was not significantly different between the LTPA and AIPA groups (524 ± 197.4 ml vs. 468.3 ± 201 ml, Mann–Whitney *U* test, *P* = 0.456). The mean hospital stay was 17.6 ± 4.7 days for patients in the AIPA group and 18.0 ± 6.2 days in the LTPA group (Mann–Whitney *U* test, *P* = 0.844) (Table 2).

**Table 2:** Comparison of surgical outcomes between AIPA and LTPA groups.

Variance	AIPA	LTPA	*p* value
Surgery time (min)	192.29 ± 34	230.47 ± 49	0.02
Blood loss (ml)	468.3 ± 201	524 ± 197.4	0.456
Duration of hospitalization	17.6 ± 4.7	18.0 ± 6.2	0.844
Cobb angle			
Pre-operative	18.71 ± 6.84	16.55 ± 7.94	0.411
Post-operative	3.98 ± 6.11	2.49 ± 6.09	0.525
Latest follow-up	3.91 ± 7.76	1.93 ± 5.32	0.542
Final loss of correction	0.74 ± 1.87	0.51 ± 2.87	0.807

Two independent samples t test or Mann-Whitney U test, Significance was set at *P* < 0.05.

### Radiological evaluation

**Figure 4:**
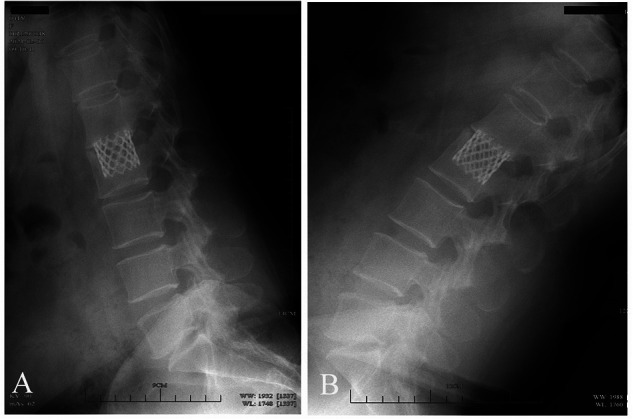
Spinal hyperextension–hyperflexion (**A,B**) A patient with L2 fracture showing good segmental mobility after removal of posterior internal fixation.

The average preoperative sagittal kyphotic angle was 18.71° ± 6.84° in the AIPA group and 16.55° ± 7.94° in the LTPA group. The postoperative average kyphotic angle was 3.98° ± 6.11°and 2.49° ± 6.09°, respectively. At the latest follow-up, the average kyphotic angle was 3.91° ± 7.76°and 1.93° ± 5.32° (AIPA and LTPA), respectively. The average loss of correction was 0.74° ± 1.87° and 0.51° ± 2.87° in the AIPA and LTPA groups, respectively, which was not significant (Mann–Whitney *U* test, *P* = 0.807) (Table 2). At the latest follow-up, all patients had solid bone fusion. Sagittal/coronal CT reconstructions showed continuous bridging callus formation. A total of 15 patients removed their posterior instrumentation at the latest follow-up. x-rays in the hyperextension–hyperflexion position showed good segmental motion with no more than 3° change according to the method described by Schnake et al (24) (Figure 4).

### Function and complication evaluation

The mean functional outcomes for ODI and SF-12 were recorded at the follow-up (Table 3). The ODI scale evaluated one month after the surgery was 43.4% ± 7.7% in the AIPA group and 60.8% ± 8.7% in the LTPA group (*P* < 0.05), but 14.7% ± 7.4% and 17.2% ± 11.8% (*P* = 0.567), respectively, at the latest follow-up (Table 3). Regarding the SF-12 evaluation, the PCS at one month after the surgery and at the latest follow-up was not significantly different between the two groups, but the MCS was 49.0% ± 5.95% in the AIPA group and 43.7% ± 8.8% in the LTPA group (*P* = 0.015) (Table 3). The mean VAS pain score improved from 8.0 ± 0.99 and 8.2 ± 1.06 in the AIPA and LTPA groups preoperatively to 2.22 ± 1.11 and 2.56 ± 1.14 postoperatively, respectively. At the latest follow-up visit, the mean VAS pain score was 0.72 ± 0.34 and 0.69 ± 0.39 in the AIPA and LTPA groups, respectively (*P *> 0.05). At the latest follow-up, 35 of the 40 patients with neurological deficits had at least one level or more of neurological recovery (Table 4).

**Table 3:** 12-Item short-form health survey and ODI.

	AIPA	LTPA	*P* value
PCS			
Post-op (1m*)	21.4% ± 3.4%	20.1% ± 3.8%	0.379
Latest follow-up	49.2% ± 3.2%	44.8% ± 10.7%	0.223
MCS			
Post-op (1m*)	49.0% ± 5.95	43.7% ± 8.8%	0.015
Latest follow-up	55.5% ± 5.8%	54.2% ± 5.3	0.475
ODI			
Pre-op	96.0% ± 3.6%	96.7% ± 3.1	0.618
Post-op (1m*)	43.4% ± 7.7%	60.8% ± 8.7%	0.000
Last follow-up	14.7% ± 7.4%	17.2% ± 11.8%	0.567

PCS, physical component score; MCS, mental component score; ODI, Oswestry Disability Index; 1m*, one month; Significance was set at *P* < 0.05.

**Table 4:** Neurological status of patients in AIPA and LTPA groups.

ASIA Impairment Grade (Admission)	ASIA Impairment Grade at Time of Latest Follow-up									
	AIPA Group					LTPA Group				
	A	B	C	D	E	A	B	C	D	E
A	1		1			1		2		
B				1					1	
C				2	6				2	4
D				1	10					8
E					13					15
The values are given as the number of patients										

ASIA, American Spinal Injury Association.

Ten patients [9 (27.2%) in the LTPA group and 1 (2.9%) in the AIPA group] experienced a temporary motor deficit in hip flexor or groin dysesthesia or thigh numbness and these symptoms were not reported preoperatively. In the LTPA group, three patients complained of hip flexion weakness, two of whom had L3 segment fractures and one had an L4 segment fracture; Five patients reported numbness or abnormal sensation in the thigh or groin area, three of whom had L2 segment fractures, one had L1 segment fracture, and one had L3 segment fracture; One patient with an L2 segment fracture reported thigh pain. In the AIPA group, one patient with an L2 segment fracture complained of numbness in the anterior thigh. The patients reported that these symptoms interfered with their lives to a greater or lesser extent and finally disappeared about 6 months after the surgery. No other complications were observed intraoperatively (Table 5).

**Table 5:** Approach-related complications.

Complications	AIPA	LTPA
Hip flexor weakness	0	3 (9.1%)
Thigh numbness	1	4 (12.1%)
Thigh pain	0	1 (3.03%)
Groin dysesthesia	0	1 (3.03%)
knee extension weakness	0	0
Vascular injuries	0	0
Urinary injury	0	0
Spinal nerve injury	0	0
Total	1 (2.9%)	9 (27.2%)

## Discussion

Open anterior thoracolumbar surgery has been gradually replaced by minimally invasive surgery (MIS) in recent years due to its high invasiveness, excessive bleeding, or complications in thoracolumbar burst fractures (8). Minimally invasive, direct LTPA has been reported for fusion in lumbar degenerative disease because of its advantages of limited exposure-related damage to soft tissues (9). Several studies showed good efficacy of the “mini-open, LTPA” in correcting kyphosis and reconstructing the anterior column in unstable thoracolumbar burst fractures (11, 23, 25). However, LTPA has some limitations of its own. The lumbar plexus is located within the psoas and releases multiple motor and sensory nerves. Hip flexion weakness may be caused by direct injury to the psoas or lumbar plexus. Allergic sensations in the groin and thighs may be caused by injury to the ilioinguinal or genitofemoral nerve (26).

The complications associated with psoas splitting included thigh symptoms (numbness, paresthesia, dysesthesias, or weakness) in 1%–8% (hip flexor weakness) and 5%–49% (sensory nerve injury) of patients undergoing lumbar fusion surgery (12, 27), because sometimes sensory nerves could not be monitored. Yilmaz reported that LTPA with corpectomy was associated with higher rates of neurological injury vs. LTPA alone (32.4% vs. 22.7%) because of acquiring larger psoas splitting for corpectomy and titanium cage placement (10). Gandhoke et al. reported that both patients with lumbar burst fractures had transitory hip flexor weakness postoperatively associated with the LTPA, but this resolved prior to discharge. Eck et al. reported that patients had pain or numbness in the left thigh postoperatively; these symptoms gradually disappeared at subsequent follow-up, but they were still not completely relieved (3, 11).

A cadaveric specimen study showed an oblique anatomical corridor: the average entry channel diameters at L2-3, 18.60 mm and 25.50 mm for static state and mild psoas retraction without psoas rupture; at L3–4, 19.25 mm and 27.05 mm; and at L4–5 for 15.00 mm and 24.45 mm, respectively (17). The most commonly used titanium cage is 22 mm in diameter, which is difficult to implant without retraction of the psoas. The AIPA procedure changes the transverse psoas approach to an oblique approach by separating the fascia of the anteroinferior border of the psoas and retracting the psoas posteriorly with a long retractor under direct visualization (16) (Figure 1B). In this study, nine patients (27.2%) reported approach-related complications in the LTPA group (three cases of hip flexor weakness and six cases of numbness or pain in the anterior thigh or groin), but all were relieved about 6 months after the surgery. One patient (2.9%) in the AIPA group had anterior thigh numbness; it was speculated that it might be caused by excessive stretching of the psoas, or by the agitation of the sensory nerves. Similarly, the rate of complications associated with AIP surgical access in 226 patients with lumbar degenerative disease was 4.9% including transient thigh pain/numbness or psoas weakness (2.2%) in the study by Fan et al (16). Hence, direct visualization and psoas retraction could reduce psoas and lumbar plexus injury in AIPA.

The mean surgery time for LTPA combined with posterior pedicle screw fixation was 230.47 ± 49 min, which was not different from the previous MIS. Machino et al. and Hu et al. reported the average surgery time of 256 min and 230 min in the combined surgery, respectively (11, 28–30). The average surgery time in the AIPA group was 192.29 ± 34 min, which was shorter than that in the LTPA group (*P* = 0.02). In the process of establishing working channel to the posterior border of the injured vertebral body, the LTP approach had to avoid lumbar plexus when directly splitting the psoas major muscle in the lumbar 3–5 segment. We often encounter larger lumbar plexus nerves obliquely crossing the lateral aspect of the vertebral body, especially with multiple nerves. Therefore, surgical manipulation can be tricky in these cases. During the decompression procedure, the surgeon may be more hesitant, which undoubtedly prolongs the surgery time and makes the procedure inconvenient. The AIP incision is more anterior than the LTP incision, and the working channel is placed obliquely. After separating the fascia below the psoas, the psoas is retracted with a special long pulling hook, which is relatively simple to manipulate. Also, the operator does not need to worry too much about lumbar plexus injury during the subsequent decompression operation. A prospective multicenter trial by Uribe et al. indicated that a prolonged psoas retraction time was a predictor of declining lumbar plexus integrity in MIS-XLIF/DLIF (extreme/direct lateral lumbar fusion) (31). In conventional open posterior/anterior combined surgery, previous studies reported an average bleeding of more than 900 ml; The value was much lower than that for the open surgery in minimally invasive surgery.

Most patients in the AIPA group showed better satisfaction than those in the LTPA group regarding the MCS and ODI scores, especially in the early postoperative phase. This might be attributed to the lower incidence of AIPA-related neurological complications. Hip flexor weakness and thigh area numbness had an impact on the patients' psychological assessment. A majority of patients improved at 6 months after the surgery, which explained the lack of differences in SF-12 and ODI assessments between the two groups at the latest follow-up. Thus, the patient's postoperative quality of life should also be taken into account, when choosing the surgical approach.

Although AIPA has some advantages in reducing approach-related complications, it faces some difficulties in managing fractures of the L4 segment, especially when the psoas is extremely hypertrophic or relatively anteriorly positioned. First, surgeons need to adequately assess the size of the space between psoas and large artery, and the thickness of psoas by preoperative axial T2-weighted MRI image at the surgical segment. The relative contraindication to AIPA in the L4 segment is that the psoas is extremely hypertrophic or more anteriorly positioned. In our experience, we placed the patient in a right lateral position with the left knee and hip flexed, as well as elevating the left lower leg by placing a pillow between the legs to release the tight psoas muscle. The AIPA procedure often required an assistant to use pulling hooks to pull the psoas major muscle posteriorly, which was laborious and unstable when the psoas was thickened. To address this challenge, after we separated the anteroinferior border of the psoas from the disc/vertebral body under direct vision and pulled posteriorly, the working channel was placed at a slightly oblique angle along the lower edge of the pulled psoas to perform lumbar corpectomy and titanium mesh placement.

Biomechanical studies showed that combined surgery resulted in better stability (32). Posterior instrumentation provided stronger stabilization and prevented further spinal canal compromise in severe burst vertebra, making the anterior monosegmental reconstruction easier. The anterolateral plate was not required in the present study to reduce the invasiveness of the surgery (23, 28). The biggest biomechanical advantage of the lateral plate was motion restriction during lateral bending. Anterolateral plate implantation *via* a mini-open corridor often required greater exposure (at least three vertebrae exposures). It also entailed increased surgery time and bleeding, which potentially made the procedure more difficult and invasive. Further, it limited the motion of the spine after the removal of the posterior instrumentation. Christiansen et al. reported that only one stand-alone lateral plate could not provide enough support compared with posterior instrumentation (33). At the latest follow-up, none of the patients experienced significant subsidence of the titanium cage in our study, although this may require a longer-term follow-up.

**Figure 5:**
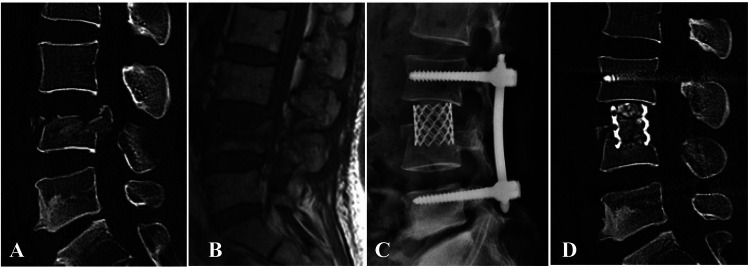
A patient with L4 burst fracture underwent posterior instrumentation combined with AIPA monosegmental reconstruction, in which the inferior endplate and disc below were preserved. (**A,B**) Preoperative sagittal CT scan and MRI images. (**C,D**) Postoperative x-ray and CT image demonstrating good correction of kyphotic deformity via posterior instrumentation combined with anterior decompression and titanium cage placement.

The integrity of the lower endplate and disc is critical to the recovery of motor function postoperatively. Hu et al. adopted preservation of the lower endplate and disc in Denis type-B burst fracture, but lacked further assessment of lumbar motion after removal of internal fixation hardware (28). The present study showed that several patients with intraoperative preservation of the inferior endplate and disc demonstrated good recovery of lumbar motion after removal of posterior instrumentation in spinal hyperextension–hyperflexion x-ray images (Figures 4, 5), although it was not assessed systematically.

Although the clinical outcome was promising, the present study also had some limitations. First, it was not a randomized controlled trial. Also, the small sample due to strict inclusion criteria did not adequately reflect the advantages and disadvantages of the technology. Finally, all results were obtained at a single center. Therefore, further studies should involve more centers to evaluate the efficiency of the novel combination procedure.

## Conclusions

Mini-open, anterior mono-segment reconstruction in unstable lumbar burst fractures *via* the AIP approach provided excellent clinical and radiographic results, with fewer approach-related complications and a shorter surgery time than the transpsoas approach. Through proper selection of indications, this modified surgical approach can be one of the options for the surgical treatment of lumbar burst fractures.

## Data Availability

The raw data supporting the conclusions of this article will be made available by the authors, without undue reservation.
